# Supplementary material for: Tailoring amorphous boron nitride for high-performance two-dimensional electronics
Source: Nat Commun. 2024 May 13;15:4016. doi: 10.1038/s41467-024-48429-4 (PMC11091059; doi:10.1038/s41467-024-48429-4)
Supplement: Supplementary file 1 — Supplementary Information [file 41467_2024_48429_MOESM1_ESM.pdf]

Supplementary Information

Tailoring Amorphous Boron Nitride for High-Performance Two-Dimensional Electronics

Cindy Y. Chen,[1] Zheng Sun,[2] Riccardo Torsi,[1] Ke Wang,[3] Jessica Kachian,[4] Bangzhi Liu,[3] Gilbert B. Rayner, Jr,[5] Zhihong Chen,[2] Joerg Appenzeller,[2] Yu-Chuan Lin,[6*] Joshua A. Robinson[1, 3, 7*]

1. Department of Materials Science and Engineering, The Pennsylvania State University, University Park, PA 16802, USA
2. School of Electrical and Computer Engineering and Birck Nanotechnology Center, Purdue University West Lafayette, IN 47907, USA
3. Materials Research Institute, The Pennsylvania State University, University Park, PA 16802, USA
4. Intel Corporation, 2200 Mission College Blvd, Santa Clara, CA 95054, USA
5. The Kurt J. Lesker Company, 1925 PA-51, Jefferson Hills, PA 15025, USA
6. Department of Materials Science and Engineering, National Yang Ming Chiao Tung University, Hsinchu City 300, Taiwan
7. Two-Dimensional Crystal Consortium, The Pennsylvania State University, University Park, PA 16802, USA

*Yu-Chuan Lin (Email: ycl194@nycu.edu.tw)
*Joshua A. Robinson (Email: jar403@psu.edu)

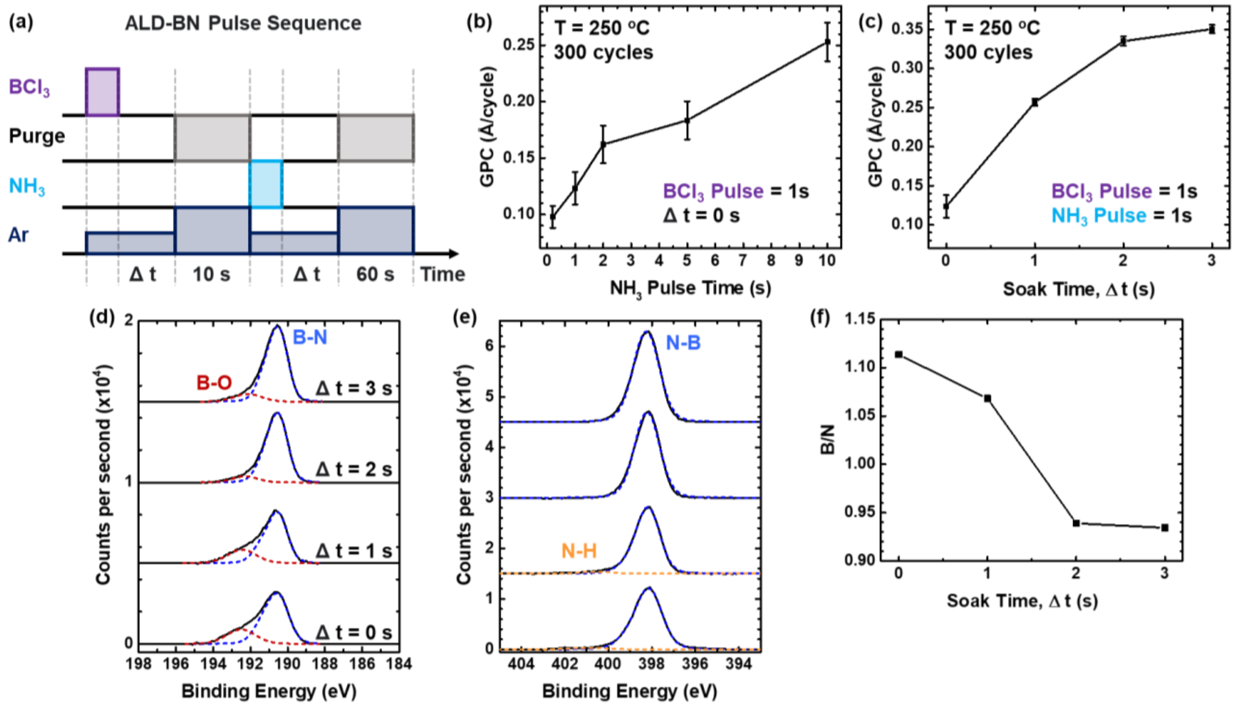

Supplementary Fig. 1: (a) Schematic of the pulse sequence in ALD of aBN. Precursor and reactant pulses are each followed by a soak step before purging, enabling saturation in aBN growth per cycle (GPC) – likely through increased adsorbate surface coverage after each half cycle - and improving wafer-scale uniformity. GPC is calculated from the average thickness of aBN deposited at 250 °C on a 150 mm Si wafer for 300 ALD cycles. Purge times after the BCl_3 and NH_3 pulse and soak steps are 10 and 60 s, respectively. (b) GPC as a function of NH_3 pulse time with no soak times. (c) GPC as a function of soak time at pulse times of 1 s for both BCl_3 and NH_3. As soak time increases, the variation in thickness across the 150 mm Si wafer decreases significantly, indicating improved wafer-scale uniformity. (d) B 1*s* and (e) N 1*s* core level XP spectra corresponding to the aBN films shown in (c). (f) Film stoichiometry B/N quantified from B 1*s* and N 1*s* spectral regions corresponding to the aBN films shown in (c).

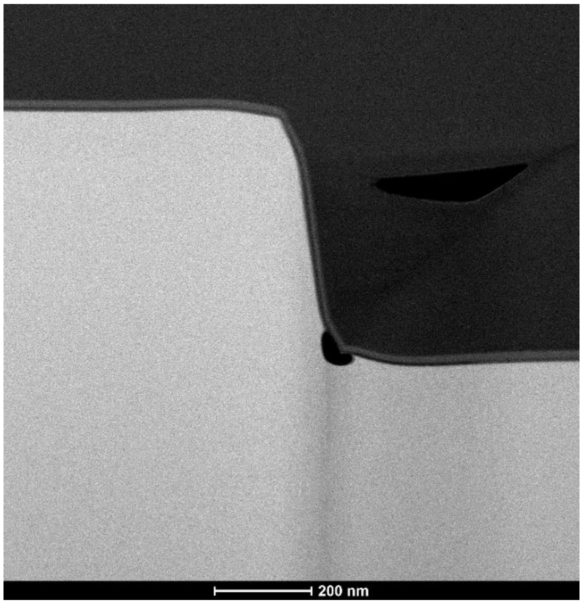

Supplementary Fig. 2: HAADF-STEM cross-sectional image of aBN deposited at 250 ℃ for 300 ALD cycles on a damascene structure made of SiO_x.

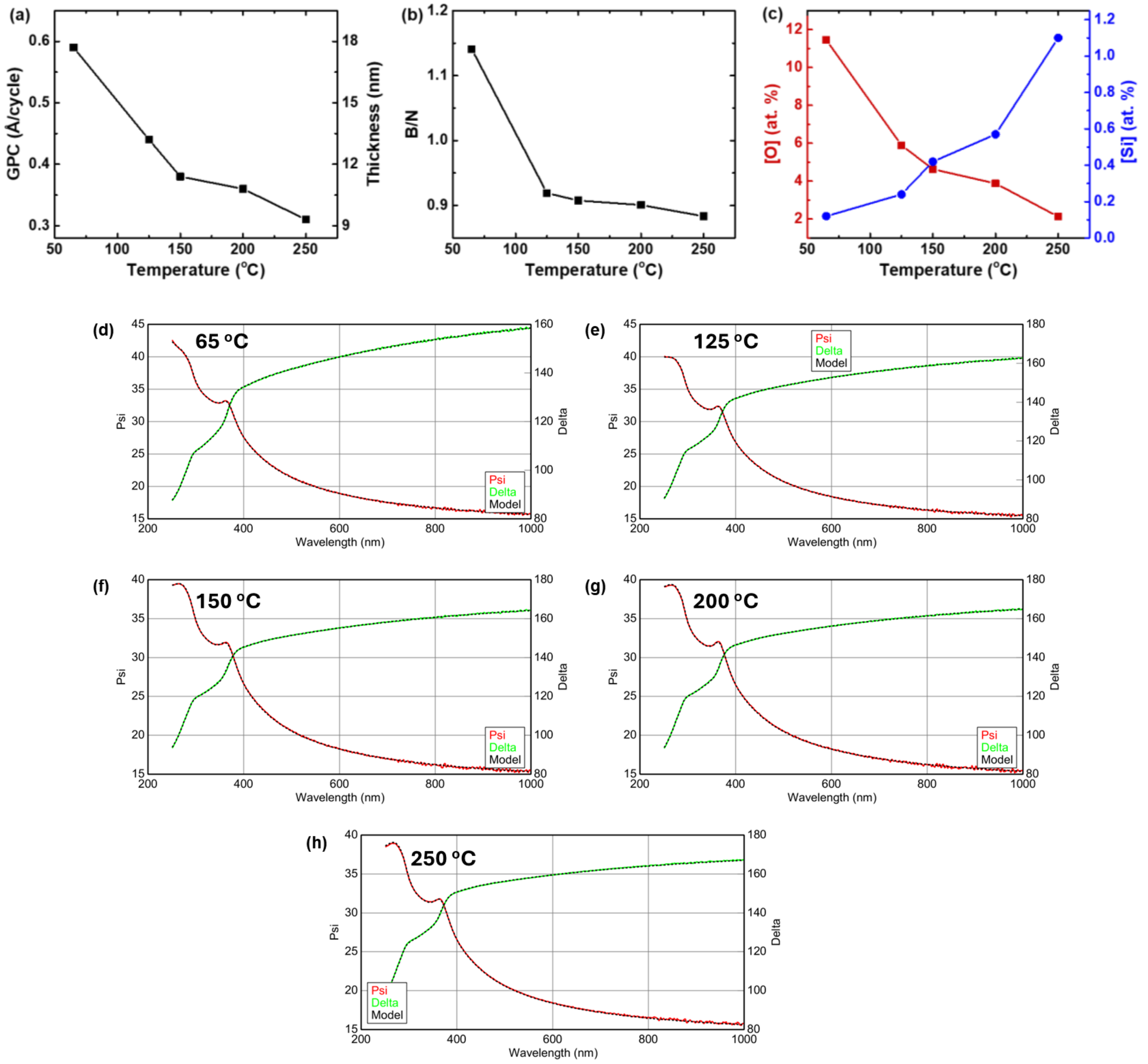

Supplementary Fig. 3: The deposition temperature dependence of aBN film properties is investigated for aBN deposited on Si wafers from 65 – 250 °C for 300 ALD cycles. (a) GPC and average thickness of aBN deposited from 65 – 250 °C. (b) B/N ratio quantified from B 1*s* and N 1*s* spectral regions in XPS of aBN films deposited from 65 – 250 °C. (c) O and Si concentrations quantified from the B-O binding energy component of the B 1*s* peak and the Si 2*p* spectral region, respectively, in XPS of aBN deposited from 65 – 250 °C. It is assumed that all oxygen within the aBN film is bound to boron. (d)-(h) Spectroscopic ellipsometry data and model fits for the thickness of aBN deposited from 65 – 250 °C.

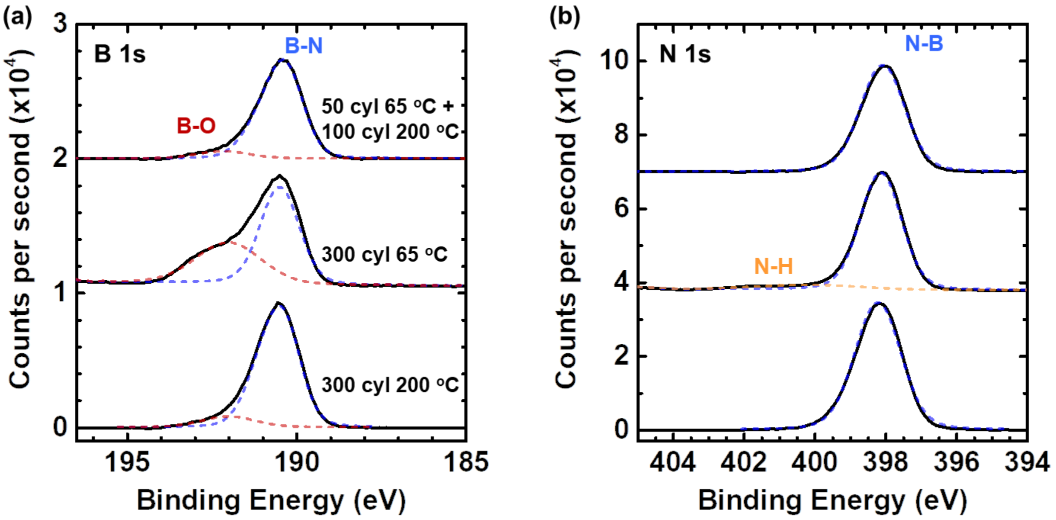

Supplementary Fig. 4: (a) B 1s and (b) N 1s core level XP spectra of aBN deposited at different temperatures on Si substrates. The pure 65 and 200 ℃ aBN films have a thickness of 17.6 and 11.2 nm, respectively. When 65 ℃ aBN is immediately followed by aBN deposition at 200 ℃ without sample removal into ambient environment, the oxidation level measured for the hybrid aBN film stack is significantly lower than that estimated for the hybrid aBN film stack using oxidation levels of the pure 65 ℃- (i.e., aBN resulting from deposition at 65 ℃ only) and pure 200 ℃- (i.e., aBN resulting from deposition at 200 ℃ only) aBN films and accounting for the 65 ℃- and 200 ℃-aBN ALD thickness contributions to the hybrid aBN film stack (see text below).

Comparison of (1) the measured O at% in the hybrid aBN film stack and (2) the estimated O at% in the hybrid aBN film stack is shown below. The estimation assumes the 65 ℃-aBN component film of the hybrid aBN film stack is not transformed by the subsequent in situ 200 ℃ aBN ALD and is oxidized ex situ (poor capping by the 200 ℃-aBN component film of the hybrid aBN film stack) and/or in situ.

(1) Measured O at% in hybrid aBN film stack = 3.0%

(2) For the hybrid aBN film stack (aBN ALD at 65 ℃ followed by aBN ALD at 200 ℃, all in situ), thickness for each component aBN layer and thickness of the hybrid aBN film stack can be calculated based on the number of ALD cycles completed for each component deposition and the GPC for aBN ALD at 65 and 200 ℃ from **Supplementary Fig. 3a**:

50 cycles * 0.59 Å/cycle = 29.5 Å (2.95 nm) of 65 ℃ aBN

100 cycles * 0.36 Å/cycle = 36 Å (3.6 nm) of 200 ℃ aBN

Total hybrid aBN film stack thickness = 2.95 nm + 3.6 nm = 6.55 nm (45% 65 ℃ aBN, 55% 200 ℃ aBN)

From **Supplementary Fig. 3c**, the O concentrations in the pure 65 ℃- (i.e., aBN resulting from deposition at 65 ℃ only) and pure 200 ℃- (i.e., aBN resulting from deposition at 200 ℃ only) aBN films are 11.4% and 3.9%, respectively. Using these O concentration values, the estimated O at% in each component film of the hybrid aBN film stack and the estimated O at% in the entire hybrid aBN film stack are as follows:

0.45 * 11.4% = 5.1% (estimated O at% in 65 ℃ aBN component of the hybrid aBN film stack)

0.55 * 3.9% = 2.2% (estimated O at% in 200 ℃ aBN component of the hybrid aBN film stack)

The estimated O at% in the entire hybrid aBN film stack = 7.3%.

The estimated (versus measured) O at% in the entire hybrid aBN film stack is > 2× higher.

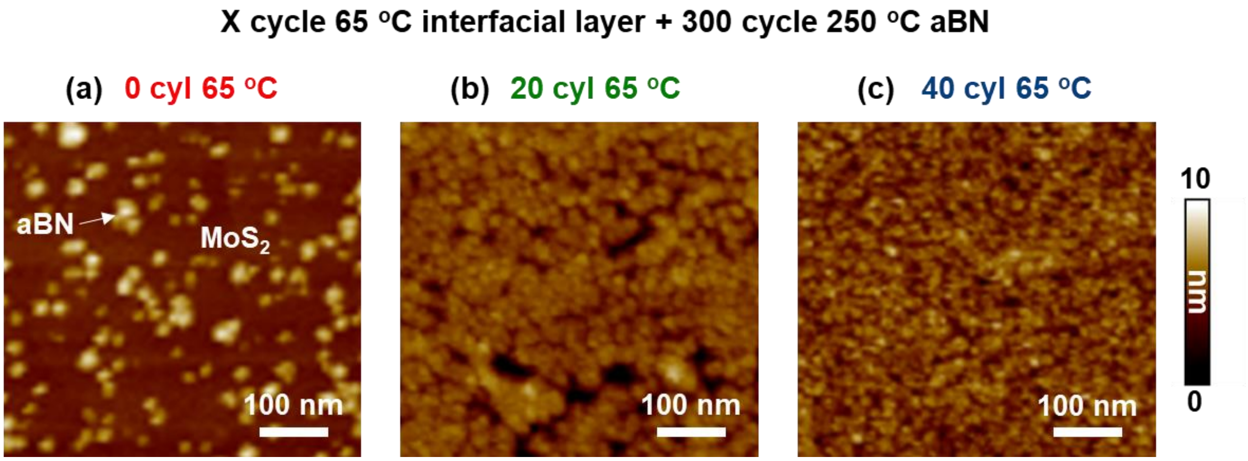

Supplementary Fig. 5: AFM image of aBN deposited on MoS_2 with (a) 0, (b) 20, and (c) 40 cycles of the 65 °C aBN interfacial layer, which is immediately followed by 300 cycles of 250 °C aBN deposition. Coalescence of the aBN film occurs when at least 40 cycles of the 65 °C interfacial layer is integrated.

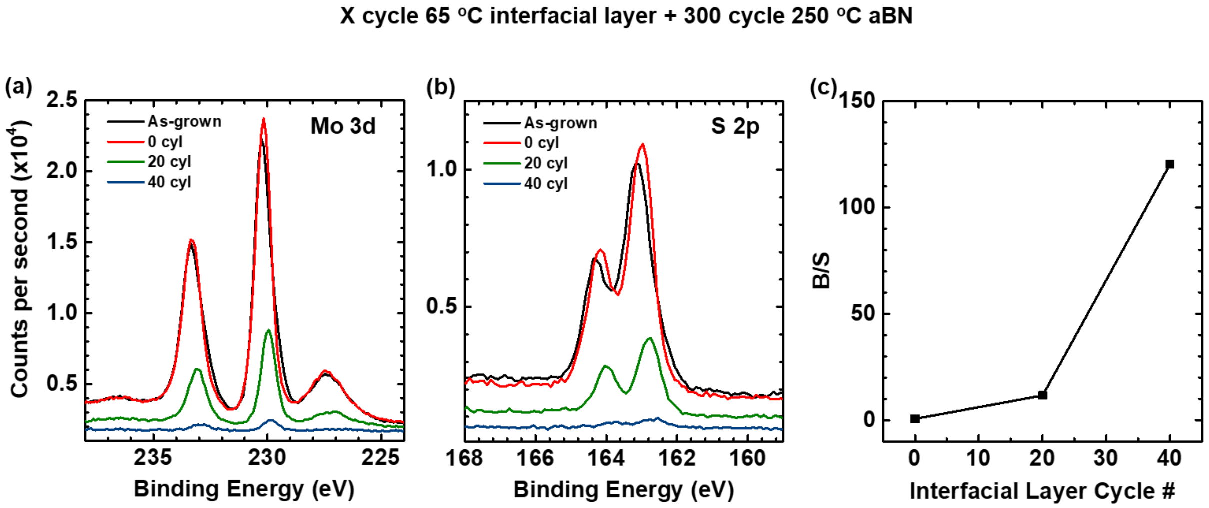

Supplementary Fig. 6: (a) Mo 3*d* and (b) S 2*p* XP spectra of as-grown and aBN-capped MoS_2. All aBN encapsulation begins with varying number of ALD cycles of the 65 °C interfacial layer, followed by 300 cycles of 250 °C aBN. (c) B/S ratio calculated from quantification of B 1*s* and S 2*p* XP spectral regions shows exponential increase in B signal with increasing interfacial layer ALD cycles, indicating enhanced nucleation of aBN at 40 cycles of the interfacial layer. B/S~0 for 0 cycles of interfacial layer highlights that significant aBN nucleation at 250 °C on MoS_2 cannot occur without the integration of an interfacial layer.

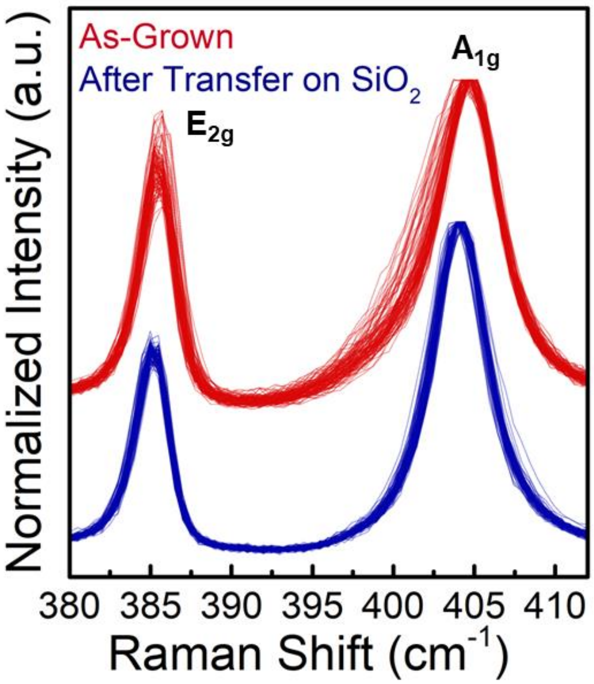

Supplementary Fig. 7: Raman spectra of as-grown and transferred monolayer MoS_2. The E_{2g} and A_{1g} peak FWHM decreases after transfer, indicating a release of the strain induced by the growth substrate.

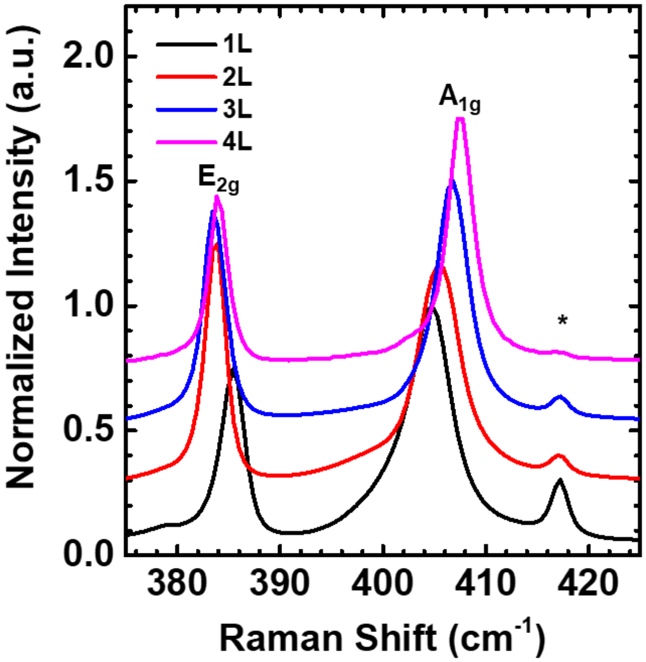

Supplementary Fig. 8: Raman spectra of as-grown 1-4L MoS_2 on c-plane sapphire. The peak separation between E_{2g} and A_{1g} increases as layer number increases. * denotes the sapphire substrate Raman peak.

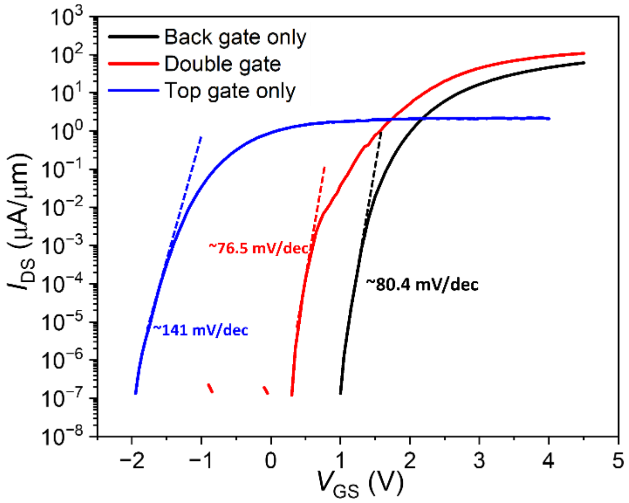

Supplementary Fig. 9: Transfer curves of a double gated ML MoS_2 FET under different gate operation modes, including back gate only sweep (top gate floating), double gate sweep with top and bottom gate connected, and top gate only sweep (back gate floating).
